# High-dimensional mediation analysis for continuous outcome with confounders using overlap weighting method in observational epigenetic study

**DOI:** 10.1186/s12874-024-02254-x

**Published:** 2024-06-03

**Authors:** Weiwei Hu, Shiyu Chen, Jiaxin Cai, Yuhui Yang, Hong Yan, Fangyao Chen

**Affiliations:** 1 Department of Epidemiology and Biostatistics, School of Public Health, Xi’an Jiaotong University, Xi’an, 710061 Shaanxi China; 2 Department of Radiology, First Affiliated Hospital of Xi’an Jiaotong University, Xi’an, 710061 Shaanxi China

**Keywords:** High-dimensional mediation model, Propensity score, Overlap weighting, Joint significant test, Composite null hypothesis

## Abstract

**Background:**

Mediation analysis is a powerful tool to identify factors mediating the causal pathway of exposure to health outcomes. Mediation analysis has been extended to study a large number of potential mediators in high-dimensional data settings. The presence of confounding in observational studies is inevitable. Hence, it’s an essential part of high-dimensional mediation analysis (HDMA) to adjust for the potential confounders. Although the propensity score (PS) related method such as propensity score regression adjustment (PSR) and inverse probability weighting (IPW) has been proposed to tackle this problem, the characteristics with extreme propensity score distribution of the PS-based method would result in the biased estimation.

**Methods:**

In this article, we integrated the overlapping weighting (OW) technique into HDMA workflow and proposed a concise and powerful high-dimensional mediation analysis procedure consisting of OW confounding adjustment, sure independence screening (SIS), de-biased Lasso penalization, and joint-significance testing underlying the mixture null distribution. We compared the proposed method with the existing method consisting of PS-based confounding adjustment, SIS, minimax concave penalty (MCP) variable selection, and classical joint-significance testing.

**Results:**

Simulation studies demonstrate the proposed procedure has the best performance in mediator selection and estimation. The proposed procedure yielded the highest true positive rate, acceptable false discovery proportion level, and lower mean square error. In the empirical study based on the GSE117859 dataset in the Gene Expression Omnibus database using the proposed method, we found that smoking history may lead to the estimated natural killer (NK) cell level reduction through the mediation effect of some methylation markers, mainly including methylation sites cg13917614 in CNP gene and cg16893868 in LILRA2 gene.

**Conclusions:**

The proposed method has higher power, sufficient false discovery rate control, and precise mediation effect estimation. Meanwhile, it is feasible to be implemented with the presence of confounders. Hence, our method is worth considering in HDMA studies.

**Supplementary Information:**

The online version contains supplementary material available at 10.1186/s12874-024-02254-x.

## Background

The analysis of the mediating effect was first proposed by Baron and Kenny (1986) [1] and was broadly applied in many scientific fields, such as psychological, sociological, and biomedical studies [2–4]. Mediation analysis has become a powerful tool to investigate the underlying mechanism of environmental exposures on health outcomes and identify the factors mediating the effect of exposures on outcomes [5]. Currently, analytical methods including the single mediator model [6, 7], multiple-mediators model [8], and high-dimensional mediation model [9] are proposed and available for researchers in many scientific fields.

With the development of advanced data collection techniques, high-dimensional data has become common in biomedical research. For example, in the epigenetic study, the Illumina Infinium HumanMethylation450 BeadChip array platform allows to measure the DNA methylation levels of roughly 480 K probes [10] and generates high dimensional data. Focusing on practical research, smoking affects lung function, and some DNA methylation sites may mediate the effect of smoking on lung function [11, 12]. To identify the significant mediators (CpG sites) between smoking and lung function, we can conduct mediation analysis in the collected high-dimensional data [9, 13, 14]. Obviously, this method can be used to identify the methylation sites mediating the association between environmental factors other than smoking and other health outcomes including some physical signs and diseases.

However, there are also some issues in high dimensional mediation analysis (HDMA), such as the curse of dimensionality, the false positive rate inflation caused by multiplicity and the confounding existing in observational research. To overcome these issues, scholars have proposed a series of statistical methods. Zhang et al. [9]. proposed the HIMA model consisting of variable screening based on sure independence screening (SIS), variable selection techniques based on minimax concave penalty (MCP) estimation and joint significance test. HIMA extends the multiple mediator framework to the high-dimensional setting by incorporating variable screening and variable selection techniques into multiple mediation analysis. The following high-dimensional mediation analysis methods also employ the generic procedure [13–16], which reduces dimensionality from high to moderate or low scale and then conducts multiple mediation test. For example, the HIMA2 procedure proposed by Perera et al. [17], which employs the SIS method based on the indirect effect of every single mediator and conducts debiased Lasso to obtain more accurate estimates, then utilizes the multiple-testing procedure proposed by James et al. [18] to control the false discovery rate. Moreover, to adjust the confounders of observational epigenetic studies, researchers tried to integrate propensity score (PS) into the high-dimensional mediation model by weighting or considering it as a covariate [14, 16], except for the classic regression adjustment.

Although many works have been made to tackle these problems, there are still some issues remaining in the dimensionality reduction and adjustment for confounders. For high dimensional mediation analysis, the previous studies don’t take confounders into account, just consider them as covariates [15, 19], such as HDMA, HIMA, and HIMA2 [5, 9, 17]. As is known to all, the multivariable model cannot adequately account for confounding effects in the presence of a large number of confounders [20]. If we only control confounding during the mediation test, but not in the dimension reduction stage, then a biased variable selection result may be obtained [14]. Thus, it is necessary to adjust confounders to improve the performance of variable selection.

To address this issue, researchers have adopted the PS-based method including PS regression adjustment (termed PSR) and classical PS weighting (also called inverse probability weighting, IPW) to adjust confounding during both stages [14]. However, the adjustment for confounders using the IPW based on PS still faces the issue of extreme weights caused by extreme PS distribution [21, 22]. To address the issue of extreme PS distribution, Li et al. [23]. proposed the overlap weighting (OW) method, which emphasizes individuals with the most overlap in their observed characteristics and is beneficial to provide a consistent estimator of the effect of exposure on outcome in the presence of extreme PS tails. OW belongs to the weighting confounding adjustment method based on PS and is gaining more popularity because of excellent statistical properties [24, 25]. However, the above OW method is only applied to traditional epidemic analysis, which needs to be extended to mediation analysis and high-dimensional data setting. Besides, most of the existing methods all hold the independent assumption between potential mediators, which is hard to ensure in high dimensional epigenetic data analysis [5, 9, 13–15, 18].

In this article, we incorporated the OW method into HIMA [9] and HIMA2 [17] models, respectively. In order to develop the accuracy of the screening of potential mediators, we modified the framework of variable screening in the original HIMA2 procedure. Eventually, we proposed the OW-based modified HIMA2 (mHIMA2) procedure for HDMA. We evaluated the performance of the proposed procedure and the existing models through simulation studies. All the above evaluations are based on the simulation study and real data application.

The rest of the article is structured as follows. In the next section, we introduced the notions, assumptions, models, and the procedure of adjustment for confounders in the high-dimensional mediation analysis model. Then, we conducted the Monte Carlo simulation study to evaluate the performance of various methods of confounding adjustment and two different mediation test approaches. Additionally, we applied the proposed method to the dataset GSE117859 in the Gene Expression Omnibus (GEO) databases and identified some DNA methylation markers that mediate the effect of smoking on the estimated natural killer (NK) cell level. Finally, we concluded the advantages and limitations of this study.

## Methods

### Model definitions

**Fig. 1:**
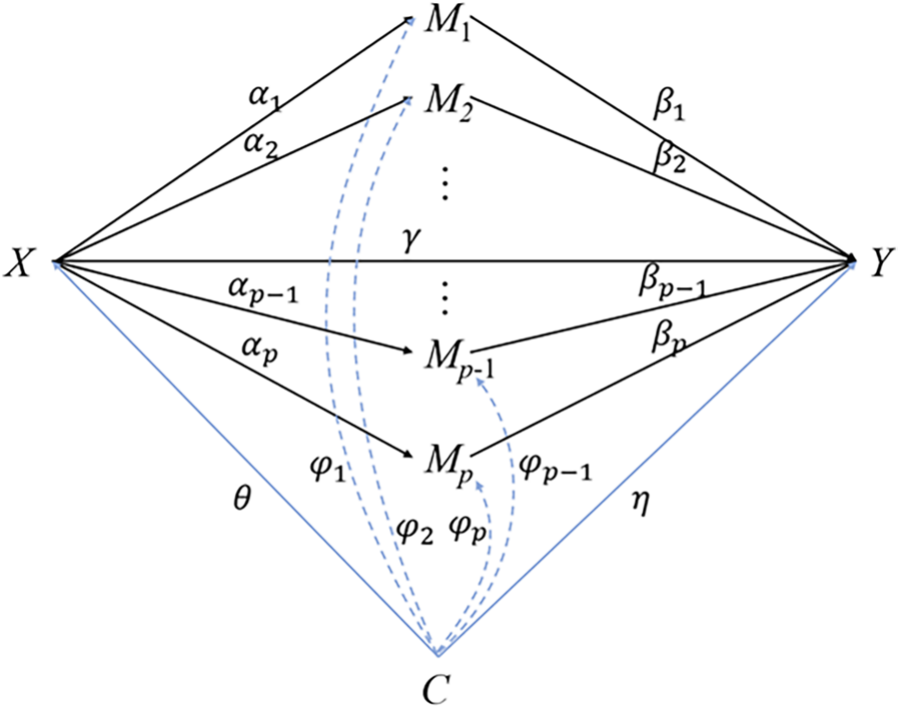
Causal diagram. High-dimensional mediation model with confounders between exposure, mediator and outcome

Our high-dimensional mediation model is shown in Fig. 1. Let $X$ be the exposure variable, where $X=1$ represents the exposed group and $X=0$ represents the controlled group. Denote the outcome as *Y*, here we mainly focus on continuous outcome. Let $M={\left({M}_{1},{M}_{2},\cdots ,{M}_{P}\right)}^{T}$ be the set of the $p$-dimensional potential mediators, where $p\gg n$, $n$ is the sample size. Let $C={\left({C}_{1},{C}_{2},\cdots ,{C}_{q}\right)}^{T}$ be the $q$-dimension baseline confounders which influence the relation of exposure-mediator, mediator-outcome, and exposure-outcome. For individual $i$, $i=\text{1,2},\cdots ,n$, we have the high-dimensional mediation models as follows:

1 $$\begin{array}{c}{M}_{ki}={a}_{k}+{\alpha }_{k}X+{{\phi }_{k}^{T}C}_{i}+{e}_{ki},k\in \left[p\right],\end{array}$$

2 $$\begin{array}{c}{Y}_{i}=a+{\gamma X}_{i}+{{\beta }^{T}M}_{i}+{\eta }^{T}{C}_{i}+{\epsilon }_{i}.\end{array}$$

where $\alpha ={\left({\alpha }_{1},{\alpha }_{2},\cdots ,{\alpha }_{p}\right)}^{T}$ is the coefficient vector relating the exposure to the mediators, $\beta ={\left({\beta }_{1},{\beta }_{2},\cdots ,{\beta }_{p}\right)}^{T}$ represents the effect of the mediators on the outcome, ${\alpha }_{k}{\beta }_{k}$ corresponds to the mediation effect of ${M}_{k}$ according to the definition of coefficients product method, and $\left[p\right]$ denotes the set of $\left\{\text{1,2},\cdots ,p\right\}$. One can consider whether ${M}_{k}$ is the statistically significant mediator or not by testing the null hypothesis ${H}_{0}:{\alpha }_{k}{\beta }_{k}=0$. ${\phi }_{k}$ and $\eta$ are the effect of $C$ on $M$ and $C$ on $Y$, respectively. ${a}_{k}$ and $a$ are the intercept term in the Eqs. 1 and 2, respectively. The same as above, ${e}_{k}$ and $\epsilon$ are each the corresponding error term. We will compare the different variable selection strategies and methods of adjusting confounders. 

### Assumptions

To ensure the identification of path-specific mediating effects, some assumptions need to be held as below. These assumptions were proposed referring to necessary condition required for high-dimensional mediation analysis suggested in published studies [8, 15, 17, 19, 26, 27]:

A1: There is no causal association between mediators. This means the proposed model contains only parallel mediators.

A2: Sequential ignorability. That consists of four assumptions listed below:

(A2.1) There are no unmeasured confounders between the exposure and the outcome;

(A2.2) There are no unmeasured confounders between the mediators and the outcome;

(A2.3) There are no unmeasured confounders between the exposure and the mediators;

(A2.4) There is no exposure-induced confounding between the mediators and the outcome.

A3: Stable unit treatment value assumption (SUTVA) [28, 29] for both the mediators and the outcome. That is to say, there is no interference between individuals.

A4: Consistency for the mediators and the outcome. That is to say, there are no measurement errors in the mediators.

A5: Positivity assumption [30]. Every individual has some positive probability of being exposed to the factor of interest.

### Proposed Procedure

We improved the HIMA procedure proposed by Zhang et al. (2016) [9] and the HIMA2 procedure proposed by Perera et al. (2022) [17] under the condition of adjusting confounders in observational data.

In this study, we developed two processes to conduct the confounding-controlled high-dimensional mediation analysis. The detailed procedure is described in the following text.

#### Step 1: PS-based methods for adjusting confounders

Since there are always some baseline confounders in observational data, we integrate propensity score (PS) into mediators (and/or outcome) models to reduce the selection bias and acquire as accurate estimates of the mediation effect as possible. Due to the PS approaches allowing the inclusion of a large scale of confounders, PS is widely used in observational research.

PS is defined as the conditional probability that a study individual with baseline covariates $C=\left({C}_{1},{C}_{2},\cdots ,{C}_{l}\right)$ would be exposed to certain study factors of interest [31]:

$$PS=P\left(X=1|{C}_{1},\cdots ,{C}_{l}\right).$$

PS can be estimated by classic multivariable statistical methods such as logistic regression [32] or by machine learning methods such as random forest (RF) and generalized boosted model (GBM) [33, 34]. In practice, logistic regression is the most commonly used. The PS of $i$th individual ${\pi }_{i}=P\left({X}_{i}=1|{C}_{1i},\cdots ,{C}_{li}\right)$ can be expressed as follows:

$$\begin{array}{c}logit\left({\pi }_{i}=P\left({X}_{i}=1\right)\right)={\theta }_{0}+{\theta }_{1}{C}_{1i}+\cdots +{\theta }_{l}{C}_{li},\end{array}$$

where $\theta ={\left({\theta }_{1},{\theta }_{2},\cdots ,{\theta }_{l}\right)}^{T}$ represents the effect of the confounders on the exposure. Then we can adopt some PS-based techniques to adjust confounders such as matching [35], stratification [36], regression [31], and weighting [37]. Here, we focus on PS regression (PSR) and PS weighting [14] (PSW, also called IPW short for inverse probability weighting) techniques to adjust potential confounders between exposure, mediators and outcome.

PSR approach incorporates PS as a covariate into the original regression model to adjust for the probability of being exposed to study factors and to reduce confounding [32]. That is similar to taking all confounders as covariates in a classical regression approach which usually uses the linear regression model for continuous outcomes and the logistic regression model for binary outcomes [38]. For the PSR approach, we can estimate the effect through the models below:

3 $$\begin{aligned} \begin{array}{ll}{M}_{ki}={a}_{k}+{\alpha }_{k}X+{{\phi }_{k}^{{\prime }}\pi }_{i}+{e}_{ki},k\in \left[p\right],\end{array}\\ \begin{array}{c}{Y}_{i}=a+{\gamma X}_{i}+{{\beta }^{T}M}_{i}+{PS}_{i}+{\epsilon }_{i}.\end{array}\end{aligned}$$

The PSW approach constructs the inverse probability weights by taking the reciprocal of PS. For binary exposure, the weight of the exposed group $X=1$ is given as $\frac{1}{PS}$, and that of the controlled group $X=0$ as $\frac{1}{1-PS}$. For $i$th individual:

$${ipw}_{i}=\frac{1}{P\left({X}_{i}=1|C\right)}=\frac{{X}_{i}}{{\pi }_{i}}+\frac{{(1-X}_{i})}{\left(1-{\pi }_{i}\right)} .$$

Then, we can estimate the coefficients of *X* in pathways $X\to M$ and $M\to Y$ by weighted estimation:

4 $$\begin{aligned} \begin{array}{ll}{M}_{ki}={a}_{k}+{\alpha }_{k,ipw}X+{{\phi }_{k}^{T}C}_{i}+{e}_{ki},k\in \left[p\right],\end{array}\\ \begin{array}{c}{Y}_{i}=a+{{\gamma }_{k,ipw}X}_{i}+{{\beta }^{T}M}_{i}+{\eta }^{T}{C}_{i}+{\epsilon }_{i},\end{array}\end{aligned}$$

where ${\alpha }_{k,ipw}$ and ${\gamma }_{ipw}$ are the weighted estimation according to the $ipw$ weight vector. However, the IPW often faces extreme PSs issue which may lead to extreme weights and result in biased estimates and excessive variance [23, 24].

The overlap weighting (OW) approach was proposed to address the issue of extreme PSs [23]. The overlap weight is given as $1-PS$ for the group $X=1$ and $PS$ for the group $X=0$. Note that, individuals with $PS$ of 0.5 make the largest contribution to the effect estimate, and individuals with $PS$ close to 0 and 1 make the smallest contribution. OW is likely to be beneficial in the presence of extreme tail weights [23, 39]. For individual $i$:

$${ow}_{i}=\left\{\begin{array}{c}1-{\pi }_{i}, { X}_{i}=1 \\ {\pi }_{i}, {X}_{i}=0 \end{array}\right. .$$

Then, the effect estimation of OW is similar to that of the PSW procedure:

5 $$\begin{aligned} \begin{array}{c}{M}_{ki}={a}_{k}+{\alpha }_{k,ow}X+{{\phi }_{k}^{T}C}_{i}+{e}_{ki},k\in \left[p\right],\end{array}\\ \begin{array}{c}{Y}_{i}=a+{{\gamma }_{k,ow}X}_{i}+{{\beta }^{T}M}_{i}+{\eta }^{T}{C}_{i}+{\epsilon }_{i},\end{array}\end{aligned}$$

In the same way, ${\alpha }_{k,ow}$ and ${\gamma }_{ow}$ are the weighted estimation using $ow$ weight vector.

#### Step 2: Confounding-controlled SIS approach for dimensionality reduction

The SIS procedure is a general technique to reduce accurately high dimensions to below sample size [40]. We adopt the SIS method to reduce dimension $p$ from ultra-high dimension to moderate scale $d=\left[\frac{2n}{\text{log}\left(n\right)}\right]$ [9, 15].

In this study, we considered two preliminary screening strategies as described in HIMA [9] and HIMA2 [17], based on the effects of $M$ on $Y$ (${\beta }_{k}$) and the indirect effect $\left|{\alpha }_{k}{\beta }_{k}\right|$ respectively. Because the indirect effects can be both positive and negative effects, to address the influence of the signs of the estimated indirect effects, the HIMA2 approach uses the absolute values of the indirect effect to obtain the size of the effect estimate regardless of the direction. This approach ensures that mediators with large effect size can be selected.

Due to the lack of screening accuracy in SIS based on indirect effects in the presence of confounders, we conducted the SIS screening based on the effects on the path $M\to Y$ controlling confounding effects using the OW approach.

In simulation, we found that it is hard to select the true mediators based on $$\left|{\alpha }_{k}{\beta }_{k}\right|$$ in the presence of confounding factors as applied in the original HIMA2 approach. So, we modified the frame of the HIMA2 method and both adopt SIS based on the effects on the path $$M\to Y$$$${\beta }_{k}$$ in the preliminary screening to select the subset of potential mediators $${M}_{SIS}=\left\{{M}_{k}:{M}_{k} \text{i}\text{s} \text{a}\text{m}\text{o}\text{n}\text{g} \text{t}\text{h}\text{e} \text{t}\text{o}\text{p} d \text{l}\text{a}\text{r}\text{g}\text{e}\text{s}\text{t} \text{e}\text{f}\text{f}\text{e}\text{c}\text{t} of {\beta }_{k}\right\}$$.

Noticing that we need to adopt a two-step weighting method [14] to estimate ${\beta }_{k}$ for the PSW and OW methods.

First, ${\gamma }_{k,w}$ can be obtained from the following sub-model:

$$\begin{array}{c}{Y}_{i}=a+{{\widehat{\gamma }}_{k,w}X}_{i}+{{\beta }_{k}M}_{ki}+{\epsilon }_{ki}\end{array}$$

where ${\widehat{\gamma }}_{k,w}$ is the estimator of ${\gamma }_{k,ow}$ or ${\gamma }_{k,ipw}$ for each ${M}_{k}$. In addition, the residual ${\widehat{e}}_{k}$ can be derived:

$${\widehat{e}}_{k}=Y-{\widehat{\gamma }}_{k,w}X.$$

Then ${\beta }_{k}$ can be estimated by regressing ${\widehat{e}}_{k}$ on ${M}_{k}$ without weighting. Through the above SIS procedure, we can identify the important mediators and achieve the goal of dimensionality reduction.

#### Step 3: Penalized estimation

According to the HIMA procedure, after the preliminary selection of candidate mediators, further variable selection can be accomplished by the penalized estimation method. Here, we adopt the MCP [41] rather than other penalty functions, since the MCP approach has the oracle property which can select the correct model with probability tending to 1 as $n\to \infty$ [15, 41, 42].

For the $d$-dimensional subset ${M}_{SIS}$, we employed the MCP-penalized estimation to further select significant mediators set ${M}_{MCP}=\left\{{M}_{k}:{\beta }_{k}\ne 0,{M}_{k}\in {M}_{SIS}\right\}$, MCP penalty function can be defined as below:

$$\begin{array}{c}{P}_{\lambda ,\delta }\left({\beta }_{k}\right)=\lambda \left[\left|{\beta }_{k}\right|-\frac{{\left|{\beta }_{k}\right|}^{2}}{2\lambda \delta }\right]I\left\{0\le \left|{\beta }_{k}\right|<\delta \right\}+\frac{{\lambda }^{2}\delta }{2}I\left\{\left|\beta \right|\ge \delta \lambda \right\}\end{array}$$

where $\lambda >0$ is the regularization parameter which can be selected by AIC or BIC, and $\delta >0$ is the tuning parameter which determines the concavity of MCP. The MCP procedure can be implemented through the R package *ncvreg* [43]. Through MCP penalty estimation, we filtered out the mediators with too weak effects by combining SIS and MCP procedures and then acquired the small number of mediators that needed to be tested. That will help to obtain more accurate effect estimates.

Following the original HIMA2 procedure, the penalized estimation adopts the de-biased Lasso method to get the estimator ${\widehat{\beta }}_{k}$ and standard error ${\widehat{\sigma }}_{{\beta }_{k}}$. The sub-model of the de-biased Lasso method can be described below:

$$\begin{array}{c}Y=a+\gamma X+{{\beta }_{SIS}^{T}M}_{SIS}+{\eta }^{T}C+\epsilon \end{array}$$

where ${\beta }_{SIS}$ denote the effects of ${M}_{k}\in {M}_{SIS}$ on $Y$. The corresponding *P*-values ${P}_{{\beta }_{k}}$ are given as:

$$\begin{array}{c}{P}_{{\beta }_{k}}=2\left\{1-{\Phi }\left(\left|{\widehat{\beta }}_{k}\right|/{\widehat{\sigma }}_{{\beta }_{k}}\right)\right\},\end{array}$$

where ${\Phi }\left(.\right)$ is the cumulative distribution function of standard normal distribution $N\left(\text{0,1}\right)$. The de-biased Lasso method can be implemented with the R package *hdi*.

#### Step 4: PS-based multiple mediation test

After MCP-based penalized estimation, we use the Joint significance test [3, 44] (termed JS-uniform) to test the mediation effect of ${M}_{k}\in {M}_{MCP}$. The Joint significance test considers the ${M}_{k}$ as a true mediator when ${\alpha }_{k}$ and ${\beta }_{k}$ is significant simultaneously. Here, ${\alpha }_{k}$ can be estimated through different confounding adjustment methods as shown in Eqs. 1, 3, 4, and 5. ${\beta }_{k}$ can be obtained using the linear regression with considering all confounders as covariates or only including PS (summary of all confounders) as a covariate.

In other words, that is based on the *P*-values for testing the path-specific effects ${H}_{0}:{\alpha }_{k}=0$ or ${H}_{0}:{\beta }_{k}=0$. The raw *P*-value for the joint significance test [3] is defined below:

$$\begin{array}{c}{P}_{raw,k}=\text{max}\left({P}_{raw,{\alpha }_{k}}, {P}_{raw,{\beta }_{k}}\right),\#\end{array}$$where ${P}_{raw,{\alpha }_{k}}$ and ${P}_{raw,{\beta }_{k}}$ are the *P*-values for testing ${H}_{0}:{\alpha }_{k}=0$ and ${H}_{0}:{\beta }_{k}=0$. ${P}_{raw,{\alpha }_{k}}$ and ${P}_{raw,{\beta }_{k}}$ can be obtained from the mediator model (e.g. Equations 1, 3, 4, and 5) and outcome model (Eq. 2), respectively.

For the multiplicity (Type I error inflation) issue in multiple mediation testing, we adopted the Benjamini–Hochberg (BH) method [45, 46] to acquire the adjusted $p$-values as below,

$$\begin{array}{c}{P}_{BH,k}=\text{min}\left({P}_{raw,k}\cdot \frac{q}{{r}_{k}},1\right),\end{array}$$

where $q$ is the number of potential mediators in the set ${M}_{MCP}$, and ${r}_{k}$ is the location number of ${P}_{raw,k}$ when all the *P*-values ${P}_{raw,k}$ are sorted ascending.

However, the Joint significance test assumes ${P}_{raw,k}$ follows a uniform null distribution. Although ${P}_{{\alpha }_{k}}$ and ${P}_{{\beta }_{k}}$ are each uniformly distributed, their maximum may not. Therefore, the Joint significance test results in a valid but overly conservative test with lower power [13, 17, 47].

Hence, we adopt the PS-based joint significance with mixture null distribution method [18] (termed JS-mixture) approach to conduct multiple mediation test after de-biased Lasso penalized estimation [17, 48] referring to the classical HIMA2 procedure. The PS-based JS-mixture approach adopts a 3-component mixture distribution as below:

$$\begin{array}{c}H_{00,k}:\alpha_k=0\;\mathrm{or}\;\beta_k\mathit=0,\;\\ H_{01,k}:\alpha_k=0\;\mathrm{or}\;\beta_k\;\neq0,\\ H_{10,k}:\alpha_k\neq0\;\mathrm{or}\;\beta_k=0.\end{array}$$

The estimated pointwise FDR for testing mediation can be computed as:

$$\begin{array}{c}\widehat{FDR}\left(t\right)=E\left[\frac{{V}_{00}\left(t\right)+{V}_{01}\left(t\right)+{V}_{10}\left(t\right)}{\text{m}\text{a}\text{x}\left(R\left(t\right),1\right)}\right],\end{array}$$

where $t\in \left[\text{0,1}\right]$, ${V}_{00}\left(t\right),{V}_{01}\left(t\right),{V}_{10}\left(t\right)$ denoting the numbers of the three types of false positives and $R\left(t\right)={V}_{00}\left(t\right)+{V}_{01}\left(t\right)+{V}_{10}\left(t\right)+{V}_{11}\left(t\right)$. The ${V}_{00}\left(t\right),{V}_{01}\left(t\right),{V}_{10}\left(t\right)$ and $\widehat{FDR}\left(t\right)$ can be obtained using the R package *HDMT*.

**Fig. 2:**
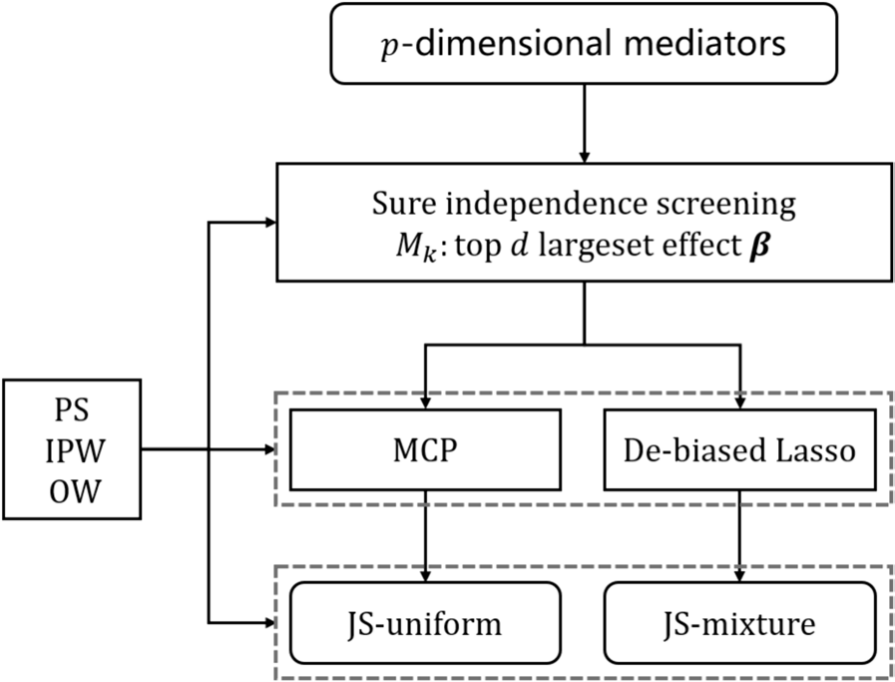
The overall workflow for high-dimensional mediation analysis under the adjusting for confounders condition

We set the significance level of 0.05 for all the tests. The detailed processes of the proposed method are summarized in Fig. 2.

## Simulation studies

### Simulation design

In this section, we conducted the simulation studies to evaluate the performance of the proposed method. The implementation of the simulation was based on R (version 4.3.0, R Foundation for Statistical Computing, Vienna, Austria) and RStudio (version 2023.9.0.463, RStudio: Integrated Development Environment for R, Boston, MA). The setting of simulation parameters was based on the published studies [9, 14, 16]. The number of replications in simulation study was set to be 500 for each combination of parameter setting referring to the replication times settings in published methodogical studies [9, 14–17, 19, 49].

The model structure is shown in Fig. 1. We consider 8 confounders $C=\left({C}_{1},{C}_{2},\cdots ,{C}_{8}\right)$ affecting the relationship of $X$, $M$, $Y$, in which continuous confounders ${C}_{1}-{C}_{4}$ follow a multivariate normal distribution $N\left(\mu ,{\Sigma }\right)$ with a mean vector $\mu ={\left(\text{0,0},\text{0,0}\right)}^{T}$ and a covariance matrix ${\Sigma }$:

$${\Sigma } = \left[\begin{array}{cccc}1& 0.3 &0.3& 0.3\\ 0.3& 1& 0.3& 0.3\\ 0.3& 0.3 & 1& 0.3\\ 0.3& 0.3 & 0.3& 1\\ \end{array}\right].$$

The last four binary confounders ${C}_{5}-{C}_{8}$ are independently generated from the Binary distribution $B\left(n,0.3\right)$, where $n$ is the sample size.

Then exposure $X$ can be generated from Binary distribution $B\left(n,{P}_{c}\right)$, where $n$ is the sample size, ${P}_{c}=1/\left(1+{e}^{-\left({\theta }^{T}C\right)}\right)$, and ${\theta }^{T}=\left({\theta }_{1},{\theta }_{2},\cdots ,{\theta }_{8}\right)=\left(\text{0.2,0.2,0.3,0.3,0.2,0.2,0.3,0.3}\right)$.

Mediators $M$ and the outcome variable $Y$ are generated according to Eqs. 1 and 2, respectively. For simplicity, we set all the effects of $C$ on $M$ to be the same. Let ${\phi }_{k}={\left({\phi }_{k1},\cdots ,{\phi }_{k8}\right)}^{T}={\left(\text{0.2,0.2,0.3,0.3,0.2,0.2,0.3,0.3}\right)}^{T}$ represent the effect of C on M. Let $\eta ={\left({\eta }_{1},{\eta }_{2},\cdots ,{\eta }_{8}\right)}^{T}={\left(\text{0.2,0.2,0.3,0.3,0.2,0.2,0.3,0.3}\right)}^{T}$ denote the effects of $C$ on $Y$.

We set the first four potential mediators ${M}_{1}-{M}_{4}$ as the true significant mediators in this study. Let $\alpha ={\left({\alpha }_{1},{\alpha }_{2},\cdots ,{\alpha }_{p}\right)}^{T}=\left(\text{0.4,0.4,0.5,0.5,0.5,0.5,0},0,\cdots ,0\right)$; $\beta ={\left({\beta }_{1},{\beta }_{2},\cdots ,{\beta }_{p}\right)}^{T}=\left(\text{0.4,0.5,0.5,0.6,0},\text{0,0.5,0.5,0},\cdots ,0\right)$. The elements of both $\alpha$ and $\beta$ are equal to zero except for the first eight elements, and the first four are the significant mediators. The mediation effect size of the true mediators ${M}_{1}-{M}_{4}$ is ${\alpha \beta }_{1-4}=\left(\text{0.16,0.2,0.25,0.3}\right)$.

Let $\gamma =0.5$; $a=0.5$; $a_k\sim U(0,1)$, $\epsilon\sim N(0,1)$. The error term ${e}_{k}$ are generated from $N\left(\text{0,1.2}\right)$ and the correlation between mediators mostly falls between 0.15 and 0.35.

To evaluate the impacts of sample size and potential mediators dimension, we set two sample size levels $n=300, 500$, and two dimension levels $p$=1000,10000.

In addition, we take the correlation between mediators into account in the condition of $p$=1000 dimension. We simulate the strong correlation between mediators by generating the error terms ${e}_{k}$ from $N\left(0,{{\Sigma }}_{e}\right)$, where ${{\Sigma }}_{e}={\left({\rho }^{\left|k-{k}^{{\prime }}\right|}\right)}_{k,{k}^{{\prime }}}$. It means the correlation between two mediators will decrease as the absolute difference in mediators’ subscript $\left|k-{k}^{{\prime }}\right|$ increases. We set four correlation levels $\rho =0, 0.25, \text{0.5,0.75}$ with dimension $p$=1000 and sample size $n=300, 500$. In the simulation setting $\rho =0, 0.25, \text{0.5,0.75}$, the corresponding Pearson correlation coefficients between two adjacent mediators are around 0.4, 0.5, 0.7, and 0.8, respectively. We evaluated the performance of the mHIMA2 and PS-based HIMA by conducting 500 replications of simulated data sets for each scenario [9, 14–17, 19, 49].

### Simulation results

Simulation results are presented in Tables 1 and 2. Evaluation of the performance of mediator selection of the proposed approach is shown in Table 1 by measuring the true positive rate (TPR) and false discovery proportion (FDP) of selection after the significance test for mediation effects. The mediators have higher TPR as the indirect effect increases (i.e., larger mediation effect, higher detection rate).

**Table 1:** TPR and FDP for the four true mediators (M1–M4)

Settings	MT methods^a^	CONF methods^b*^	TPR				Overall TPR	FDP
			M1$\alpha \beta$= 0.16	M2$\alpha \beta$= 0.20	M3$\alpha \beta$= 0.25	M4$\alpha \beta$= 0.30		
*n* = 300, *p* = 1000	HIMA	RA	0.2340	0.2740	0.4820	0.4880	0.3695	0.0027
		PSR	0.1980	0.2260	0.4380	0.4340	0.3240	0.0031
		IPW	0.2000	0.2060	0.3760	0.4120	0.2985	0.0050
		OW	0.1860	0.2000	0.4140	0.4080	0.3020	0.0033
	mHIMA2	RA	0.3298	0.3510	0.6004	0.5962	0.4693	0.0144
		PSR	0.4754	0.5246	0.7295	0.7520	0.6204	0.0670
		IPW	0.5071	0.5253	0.7333	0.7394	0.6263	0.0909
		OW	0.5396	0.5375	0.7546	0.7789	0.6526	0.0905
*n* = 300, *p* = 10,000	HIMA	RA	0.2660	0.2680	0.4540	0.4940	0.3705	0.0185
		PSR	0.2160	0.2280	0.4040	0.4460	0.3235	0.0046
		IPW	0.2020	0.1820	0.3640	0.4080	0.2890	0.0120
		OW	0.1960	0.2100	0.3840	0.4180	0.3020	0.0082
	mHIMA2	RA	0.3550	0.3203	0.5649	0.5758	0.4540	0.0141
		PSR	0.4714	0.4857	0.6776	0.6918	0.5816	0.0539
		IPW	0.5051	0.5030	0.6707	0.7131	0.5980	0.0800
		OW	0.5466	0.5344	0.7328	0.7672	0.6452	0.0834
*n* = 500, *p* = 1000	HIMA	RA	0.5440	0.5860	0.8380	0.8740	0.7105	0.0007
		PSR	0.5140	0.5420	0.8160	0.8480	0.6800	0.0022
		IPW	0.4540	0.4480	0.7280	0.7420	0.5930	0.0025
		OW	0.4800	0.4880	0.7680	0.7880	0.6310	0.0024
	mHIMA2	RA	0.6579	0.6984	0.8927	0.9170	0.7915	0.0076
		PSR	0.8060	0.8180	0.9400	0.9540	0.8795	0.0487
		IPW	0.8377	0.8297	0.9198	0.9599	0.8868	0.0679
		OW	0.8620	0.8520	0.9480	0.9760	0.9095	0.0629
*n* = 500, *p* = 10,000	HIMA	RA	0.5440	0.5640	0.8460	0.8360	0.6975	0.0050
		PSR	0.5160	0.5360	0.8220	0.8020	0.6690	0.0000
		IPW	0.4140	0.4480	0.7280	0.7220	0.5780	0.0026
		OW	0.4520	0.4900	0.7920	0.7720	0.6265	0.0016
	mHIMA2	RA	0.6451	0.6326	0.8852	0.8664	0.7573	0.0109
		PSR	0.8116	0.8196	0.9519	0.9479	0.8828	0.0657
		IPW	0.8096	0.8236	0.9419	0.9339	0.8773	0.0774
		OW	0.8417	0.8717	0.9760	0.9559	0.9113	0.0667

^a^ MT methods denote two different mediation test approaches, including HIMA Zhang et al. and modified HIMA2 Perera et al. (termed mHIMA2)

^b^ CONF methods denote different confounding adjustment methods

^*^ RA denotes regression adjustment. PSR denotes propensity score regression adjustment. IPW denotes inverse probability weighting. OW denotes overlapping weighting

**Table 2:** Estimation results of mediation effects, expressed as Mean (MSE)

Settings	MT methods^a^	CONF methods^b*^	M1 $\alpha \beta$= 0.16 (MSE)	M2 $\alpha \beta$= 0.20 (MSE)	M3 $\alpha \beta$= 0.25 (MSE)	M4 $\alpha \beta$= 0.30 (MSE)
*n* = 300, *p* = 1000	HIMA	RA	0.1547 (0.0043)	0.1909 (0.0067)	0.2414 (0.0065)	0.2907 (0.0084)
		PSR	0.1628 (0.0048)	0.1987 (0.0072)	0.2520 (0.0073)	0.3014 (0.0088)
		IPW	0.1581 (0.0054)	0.1967 (0.0072)	0.2451 (0.0071)	0.2976 (0.0094)
		OW	0.1573 (0.0044)	0.1947 (0.0069)	0.2454 (0.0065)	0.2955 (0.0083)
	mHIMA2	RA	0.1488 (0.0041)	0.1813 (0.0062)	0.2320 (0.0062)	0.2764 (0.0081)
		PSR	0.1487 (0.0042)	0.1850 (0.0064)	0.2337 (0.0063)	0.2837 (0.0083)
		IPW	0.1524 (0.0052)	0.1897 (0.0068)	0.2367 (0.0069)	0.2884 (0.0094)
		OW	0.1522 (0.0043)	0.1879 (0.0064)	0.2369 (0.0064)	0.2871 (0.0083)
*n* = 500, *p* = 1000	HIMA	RA	0.1596 (0.0024)	0.2016 (0.0038)	0.2458 (0.0040)	0.3040 (0.0046)
		PSR	0.1684 (0.0027)	0.2110 (0.0044)	0.2563 (0.0044)	0.3158 (0.0053)
		IPW	0.1607 (0.0026)	0.2024 (0.0041)	0.2470 (0.0045)	0.3051 (0.0052)
		OW	0.1594 (0.0024)	0.2019 (0.0038)	0.2459 (0.0041)	0.3041 (0.0046)
	mHIMA2	RA	0.1540 (0.0022)	0.1936 (0.0037)	0.2349 (0.0040)	0.2902 (0.0043)
		PSR	0.1548 (0.0023)	0.1966 (0.0037)	0.2392 (0.0040)	0.2977 (0.0045)
		IPW	0.1569 (0.0025)	0.1980 (0.0040)	0.2413 (0.0044)	0.2994 (0.0050)
		OW	0.1556 (0.0023)	0.1976 (0.0038)	0.2402 (0.0040)	0.2986 (0.0045)
*n* = 300, *p* = 10,000	HIMA	RA	0.1217 (0.0045)	0.1514 (0.0061)	0.1873 (0.0088)	0.2278 (0.0119)
		PSR	0.1472 (0.0044)	0.1823 (0.0050)	0.2256 (0.0064)	0.2750 (0.0087)
		IPW	0.1569 (0.0048)	0.1935 (0.0058)	0.2363 (0.0066)	0.2887 (0.0086)
		OW	0.1567 (0.0043)	0.1929 (0.0051)	0.2358 (0.0061)	0.2875 (0.0078)
	mHIMA2	RA	0.1259 (0.0039)	0.1540 (0.0053)	0.1896 (0.0076)	0.2307 (0.0102)
		PSR	0.1350 (0.0039)	0.1698 (0.0049)	0.2089 (0.0064)	0.2565 (0.0083)
		IPW	0.1425 (0.0043)	0.1780 (0.0055)	0.2169 (0.0066)	0.2692 (0.0086)
		OW	0.1425 (0.0039)	0.1780 (0.0049)	0.2170 (0.0062)	0.2670 (0.0080)
*n* = 500, *p* = 10,000	HIMA	RA	0.1502 (0.0022)	0.1911 (0.0037)	0.2364 (0.0040)	0.2836 (0.0055)
		PSR	0.1596 (0.0024)	0.2016 (0.0037)	0.2497 (0.0038)	0.2984 (0.0050)
		IPW	0.1577 (0.0023)	0.2008 (0.0038)	0.2502 (0.0037)	0.2985 (0.0052)
		OW	0.1573 (0.0021)	0.2008 (0.0034)	0.2495 (0.0035)	0.2991 (0.0047)
	mHIMA2	RA	0.1287 (0.0024)	0.1630 (0.0037)	0.2022 (0.0046)	0.2420 (0.0067)
		PSR	0.1402 (0.0021)	0.1805 (0.0032)	0.2243 (0.0035)	0.2719 (0.0048)
		IPW	0.1461 (0.0022)	0.1872 (0.0036)	0.2333 (0.0035)	0.2800 (0.0052)
		OW	0.1456 (0.0020)	0.1871 (0.0031)	0.2324 (0.0033)	0.2808 (0.0047)

^a^ MT methods denote two different mediation test approaches, including HIMA and modified HIMA2 (termed mHIMA2).

^b^ CONF methods denote different confounding adjustment methods

^*^ RA denotes regression adjustment. PSR denotes propensity score regression adjustment. IPW denotes inverse probability weighting. OW denotes overlapping weighting

As presented in Table 1. Under most settings, the mHIMA2 mediation test approach has a higher TPR than PS-based HIMA while a higher FDP at the same time. Overall, the mHIMA2 is more powerful than the PS-based HIMA and is less conservative in selecting significant mediators.

As shown in Table 1, for the mHIMA2 mediation test approach, TPR is ranked as OW > IPW > PSR > RA, and FDP is not more than 0.1 and gradually decreases to close to 0.05 as the sample size increases. Among all models, the mHIMA2 mediation test approach with OW adjustment has the highest power and acceptable false positive level. When using the PS-based HIMA mediation test approach, TPR is ranked consistently as RA > PSR > OW > IPW, and all four models also keep FDP at an extremely low level.

Table 2 presents the estimation of mediation effects with the mean and mean square error (MSE). The estimators approach the true values as the mediation effect increases. All models tend to be more accurate as $n$ gets larger and $p$ gets smaller. Overall, the mHIMA2 mediation test approach has a smaller MSE than the PS-based HIMA approach in most cases. RA adjustment has a higher MSE than other adjustment methods especially when facing the large mediation effect, OW adjustment has the lower MSE among the four adjustment methods.

As shown in Table 2, similarly, the mHIMA2 approach with OW adjustment has the smallest MSE among all models. Moreover, similar results can be seen in the different strong correlation settings in Table S1-S8 in the supplementary file. The mHIMA2 methods have lower MSE (i.e. more precise estimation) and apparently higher TPR. That means the de-biased Lasso technique in mHIMA2 methods performs better when handling the moderate correlation between mediators. However, the FDP of all models slightly increases as the correlation between mediators increases. When correlation among the mediators is strong (for example, $r$>0.7), all models suffer in terms of increased MSE.

### Data application

Smoking is an important environmental factor affecting the immune system and blood cell composition [50, 51]. Previous studies have demonstrated smokers had lower natural killer (NK) cell counts and activity [50, 51]. Smoking has also been found to be associated with DNA methylation levels [52]. Meanwhile, DNA methylation levels have also been found to be associated with associated with human NK cell activation [53, 54]. Therefore, DNA methylation may mediate the association between smoking and NK cell level. So we implemented the proposed high-dimensional mediation analysis methods to identify the specific functional CpG sites that may mediate the relationship between smoking and the estimated NK cell level.

Here we apply our method to the GSE117859 dataset obtained from the Gene Expression Omnibus (GEO) database. The aim of the study in which GSE117859 was originally measured is to explore the smoking-associated DNA methylation features linked to AIDS outcomes in the HIV-positive population [55]. The blood samples from the Veteran Aging Cohort Study (VACS) were collected in that study. The HumanMethylation450 BeadChip platform was used to measure the DNA methylation levels.

In total 608 samplesand 485,577 probes were included in the dataset. Clinical information such as age, sex, race, smoking history, adherence of antiretroviral therapy (ART), estimated CD4 T cells, estimated CD8 T cells, and estimated NK cells were collected. The estimated CD4/CD8/NK were obtained using a methylation-based cell type deconvolution algorithm proposed by Housman et al. [56]. To some extent, the estimated CD4 and CD8 levels can represent AIDS severity.

Smoking status was collected based on self-report. All included patients were classified into the smoker and the non-smoker groups according to their reported smoking history. After removing the individuals without available clinical information and DNAm sites with missing values, a total of 587 samples and 485,503 probes were included in the analysis.

We adjusted the potential confounders including age, race, adherence of antiretroviral therapy, estimated CD4 T cells, and estimated CD8 T cells. Demographic and clinical variables included in our analysis are presented in Table 3.

**Table 3:** Baseline characteristics of the HIV-positive patients included in the analysis

Variable		Non-smoker (*N* = 236)	Smoker (*N* = 351)	Total (*N* = 587)	*P*-value
Age		49.72 ± 8.86	49.13 ± 6.69	49.37 ± 7.63	0.391
Race	White	197 (83.5%)	307 (87.5%)	504 (85.9%)	0.008
	Black	21 (8.9%)	36 (10.3%)	57 (9.7%)	
	Others	18 (7.6%)	8 (2.3%)	26 (4.4%)	
ART^a^	Yes	185 (78.4%)	273 (77.8%)	458 (78%)	0.941
	No	51 (21.6%)	78 (22.2%)	129 (22%)	
CD4		0.05 ± 0.05	0.05 ± 0.05	0.05 ± 0.05	0.228
CD8		0.17 ± 0.08	0.18 ± 0.08	0.18 ± 0.08	0.368
NK		0.09 ± 0.06	0.07 ± 0.05	0.07 ± 0.06	< 0.001

^a^ *ART* adherence of antiretroviral therapy

The analysis results using the proposed mHIMA2 method are presented in Table 4. Here, we mainly presented the CpGs mediators with a total effect proportion greater than 5%. Due to the limitation of text content, we didn’t present the whole summary results of the PS-based HIMA method, but that can be seen in Table S9 in the supplementary file.

**Table 4:** Summary of the selected CpGs mediators with a %TE > 5 by the mHIMA2 models

Method^a^	CpG	Chrom	Gene	$\widehat{\alpha }$	$\widehat{\beta }$	%TE^b^	*P*-value
mHIMA2-RA	cg20460771	1	PTAFR	0.0211	-0.0572	5.2398	0.0019
	cg06040872	17	CCL18	0.0157	-0.1092	7.4323	0.0002
	cg16893868	19	LILRA2	-0.0155	0.1015	6.8344	< 0.0001
mHIMA2-PSR	cg13917614	17	CNP	0.0230	-0.1936	19.3678	0.0001
	cg03164561	2	NMUR1	0.0149	-0.1146	7.4161	0.0015
	cg03605454	4	RP11-526A4.1	0.0154	-0.1033	6.9094	0.0028
	cg09529165	17	-	0.0198	-0.2038	17.5660	< 0.0001
	cg01500140	19	LIM2	0.0157	-0.1393	9.5028	0.0013
	cg16893868	19	LILRA2	-0.0155	0.1117	7.5217	0.0012
mHIMA2-IPW	cg13917614	17	CNP	0.0230	-0.0717	7.1289	0.0001
	cg16893868	19	LILRA2	-0.0157	0.0913	6.1962	0.0001
mHIMA2-OW	cg13917614	17	CNP	0.0230	-0.0737	7.3785	0.0001
	cg16893868	19	LILRA2	-0.0155	0.0916	6.1681	0.0001

^a^Method denotes the combination of the mHIMA2 approach and different confounding adjustment methods. Such as mHIMA2-OW denotes the mHIMA2 method with overlapping weighting

^b^%TE total effect proportion

- No related genes were found

As shown in Table 4, we identified two methylation sites cg13917614 in CNP gene and cg16893868 in LILRA2 gene by most of mHIMA2 based methods. The similar result can be seen in Table S9 in the supplementary file. The existing studies have already demonstrated the site cg13917614 is associated with smoking [52, 57]. Although we don’t find direct evidence that the CNP gene is associated with immune function based on the existing literature, relevant studies showed a link between CNP and inflammatory responses in which the mechanism remains further study [58, 59].

The encoded protein of the LILRA2 gene can suppress innate immune response [60, 61]. The results reveal that smoking will promote the demethylation of cg16893868, leading to an increase in gene LILRA2 expression and ultimately reducing the estimated NK cell level. It has been found that the remaining CpG sites cg20460771, cg03164561, cg03605454, cg09529165, and cg01500140 are all associated with smoking [11, 52, 62–64]. Further insights into the discovered CpG mediators in genome-wide epigenetic studies will be meaningful.

## Discussion

The causal relationship obtained in high-dimensional mediation analysis usually depends on no-confounding assumption. However, confounding is almost inevitable in observational studies owing to the lack of randomization of the baseline covariates in practice. Previous studies show the utilization of PS method such as PS-adjustment and IPW in high-dimensional mediation analysis, but those face the issue of extreme PS distribution.

In this article, we integrated OW approach into the high-dimensional mediation model, which can address extreme PS distribution and better adjust for confounding. Finally, we developed a high-dimensional mediation analysis workflow consisting of OW confounding adjustment, SIS, de-biased Lasso penalization for potential mediator screening, and the high-dimensional mediation test underlying the mixture null distribution of *P*-values.

Simulation results indicate that the mHIMA2 with OW approach presented in this study performs best among all the compared models with the highest TPR, acceptable FDP level, and the smallest MSE in mediating effect estimation. In addition, the mHIMA2 embedded de-biased Lasso method performs better when moderate correlations between mediators exist.

Simulation study also suggestedthe proposed method would perform better when the sample size was increased. This result suggests that when the proposed method is used for the analysis of mediating effects on real data, a sufficient sample size should also be ensured. Such a feature is also consistent with other existing methods [5, 9, 14, 17, 19, 49]. Furthermore, the dimensionality of potential mediators has little effect on the performance of the proposed method.

In most of the previous studies [5, 9, 13, 17], it didn’t take confounding adjustment into account in the SIS process. However, we adopted the PS-based method to adjust confounding, thus improving the accuracy of mediators screening. Moreover, it has been assumed that mediators are linearly independent of each other, but such an assumption is often not strictly valid in real data. The violation of the mediators’ independence assumption often affects the accuracy of mediators selection and precision of mediating effect estimation. The proposed method can effectively deal with this issue which can tolerate the correlation between the mediators and ensure the robustness of mediators selection, multiple mediation testing, and mediating effect estimation.

Similar to other two-step approaches, the error of the first model may be introduced and cumulated in the second step, because the first-step can not quarantee 100% correctness. To avoid this, we set a relatively loose screening criterion with $d=2n/log(n)$ to select the top $d$ largest effect mediators [15–17, 49] in the first step to control false negative while avoiding the increase of false positive error according to the application recommendation of SIS approach. Though the errors cannot be totally avoid, this can reduce the error in the preliminary screening of mediators and prevent serious error cumulation in the second step to some extend. As shown in the simulation, the proposed two-step model performed well. Besides, previous published studies also have demonstrated the error cumulation issue in two-step models can be controlled well in the similar way as we did, and well not cause serious bias in the final results [14, 65–70].

Meanwhile, we applied the proposed method to the dataset GSE117859 obtained from the GEO databases and identified several significant DNAm mediators, including the sites cg13917614, cg16893868, cg20460771, cg03164561, cg03605454, cg09529165, and cg01500140. Among them, site cg16893868 in LILRA2 gene has been demonstrated to be associated with smoking and immune function [60, 61]. That indicates that the proposed method can identify reliable mediators in empirical data analysis.

The presence of confounding in observation studies always is a major challenge to obtaining causal relationships. Currently, most genetic studies are based on observational research without randomization of baseline characteristics. Particularly, the high-dimensional mediation analysis always faces some issues, such as the accuracy of the high-dimensional mediation selection and the low power of multiple mediation test [13, 14, 17, 18]. Although the utilization of PSR and IPW offers a solution of confounding adjustment in classical HDMA workflow, it still faces the issue of extreme PS distribution.

The proposed OW-based method can provide a more precise and stable mediating effect estimation. However, the misspecification of the outcome model and PS model can not be avoid in practice. Hence, the doubly robust methods may be desirable to be applied in HDMA workflow in future study. Even if the JS-mixture method was proposed to improve the power of multiple mediation testing, other more powerful test methods still are appealing in large-scale genome-wide epigenetic studies [13, 18]. Conducting further simulation and methodology studies to compare different powerful test methods may provide useful reference for future studies. It should also be noticed that the existence of unmeasured confounding is out of the scope of this paper. Previews published studies have provided serval applicable methods to deal with this issue [49, 71].

## Conclusion

Overall, the mHIMA2 with OW adjustment has sufficient power in selecting potential true mediators and obtaining precise estimation for mediation effects. It can be recommended in practical high-dimensional mediation analysis, especially in epigenetic study.

### Supplementary Information

Additional file 1: High-dimensional mediation analysis for continuous outcome with confounders using overlap weighting method in observational epigenetic study. Simulation results of different correlation levels  $\rho$=0,0.25,0.5,0.75 with dimension $\rho$=1000 and sample size $n$=300,500 were presented in the Table S1-8. Analysis result using the PS-based HIMA methods was shown in Table S9.

## Data Availability

The dataset GSE117859 obtained from GEO database in our real data analysis can be accessed (at https://www.ncbi.nlm.nih.gov/geo/query/acc.cgi? acc=GSE117859) without limitation. Our procedure is implemented using the R software. The corresponding R code can be found at https://github.com/huww1998/CONF_mHIMA2.

## Abbreviations

FDP: False discovery proportion
GEO: Gene Expression Omnibus
HDMA: High-dimensional mediation analysis
IPW: Inverse probability weighting
JS-uniform: Joint significant test with uniform distribution
JS-mixture: Joint significance test with mixture null distribution
MSE: Mean square error
mHIMA2: Modified HIMA2 model
NK cell: Natural killer cell
OW: Overlapping weighting
PSR: Propensity score regression adjustment
PS: Propensity score
SIS: Sure independence screening
TPR: True positive rate
