# Supplementary material for: High-dimensional mediation analysis for continuous outcome with confounders using overlap weighting method in observational epigenetic study
Source: BMC Med Res Methodol. 2024 Jun 3;24:125. doi: 10.1186/s12874-024-02254-x (PMC11145821; doi:10.1186/s12874-024-02254-x)
Supplement: Supplementary file 1 — Additional file 1: High-dimensional mediation analysis for continuous outcome with confounders using overlap weighting method in observational epigenetic study. Simulation results of different correlation levels $\rho$ρ=0,0.25,0.5,0.75 with dimension $\rho$ρ=1000 and sample size $n$n=300,500 were presented in the Table S1-8. Analysis result using the PS-based HIMA methods was shown in Table S9. [file 12874_2024_2254_MOESM1_ESM.docx]

High-dimensional mediation analysis for continuous outcome with confounders using overlap weighting method in observational epigenetic study

Table S1. TPR and FDP for the four true mediators (M1–M4), $\rho=0,p=1000$.

| Sample size | MT method^a^ | CONF method^b^ | TPR | | | | Overall TPR | FDP |
| --- | --- | --- | --- | --- | --- | --- | --- | --- |
|  |  |  | M1 $\alpha\beta$= 0.16 | M2 $\alpha\beta$= 0.20 | M3 $\alpha\beta$= 0.25 | M4 $\alpha\beta$= 0.30 |  |  |
| n=300 | HIMA | RA | 0.4740 | 0.4880 | 0.7520 | 0.7720 | 0.6215 | 0.0056 |
|  |  | PSR | 0.4100 | 0.4180 | 0.6900 | 0.7020 | 0.5550 | 0.0027 |
|  |  | IPW | 0.3160 | 0.3280 | 0.5760 | 0.5700 | 0.4475 | 0.0033 |
|  |  | OW | 0.3460 | 0.3540 | 0.6240 | 0.6040 | 0.4820 | 0.0031 |
|  | mHIMA2 | RA | 0.5464 | 0.5896 | 0.8207 | 0.8294 | 0.6965 | 0.0046 |
|  |  | PSR | 0.7540 | 0.7863 | 0.9456 | 0.9456 | 0.8579 | 0.0684 |
|  |  | IPW | 0.7117 | 0.7238 | 0.8931 | 0.9052 | 0.8085 | 0.0787 |
|  |  | OW | 0.7375 | 0.7856 | 0.9419 | 0.9359 | 0.8502 | 0.0681 |
| n=500 | HIMA | RA | 0.8060 | 0.8420 | 0.9700 | 0.9740 | 0.8980 | 0.0017 |
|  |  | PSR | 0.7720 | 0.8180 | 0.9640 | 0.9640 | 0.8795 | 0.0011 |
|  |  | IPW | 0.6020 | 0.6680 | 0.8680 | 0.8920 | 0.7575 | 0.0026 |
|  |  | OW | 0.6460 | 0.7080 | 0.9280 | 0.9380 | 0.8050 | 0.0012 |
|  | mHIMA2 | RA | 0.8636 | 0.8822 | 0.9876 | 0.9876 | 0.9303 | 0.0050 |
|  |  | PSR | 0.9680 | 0.9520 | 1.0000 | 1.0000 | 0.9800 | 0.0604 |
|  |  | IPW | 0.9180 | 0.9020 | 0.9800 | 0.9880 | 0.9470 | 0.0535 |
|  |  | OW | 0.9560 | 0.9400 | 0.9980 | 0.9960 | 0.9725 | 0.0554 |

^a^ MT methods denote two different mediation test approaches, including HIMA and modified HIMA2 (termed mHIMA2).

^b^ CONF methods denote different confounding adjustment methods.

^*^ RA denotes regression adjustment. PSR denotes propensity score regression adjustment. IPW denotes inverse probability weighting. OW denotes overlapping weighting.

Table S2. Estimation results of mediation effects, expressed as Mean (MSE), $\rho=0,p=1000$.

| Sample size | MT method^a^ | CONF method^b^ | M1 $\alpha\beta$= 0.16 (MSE) | M2 $\alpha\beta$= 0.20 (MSE) | M3 $\alpha\beta$= 0.25 (MSE) | M4 $\alpha\beta$= 0.30 (MSE) |
| --- | --- | --- | --- | --- | --- | --- |
| n=300 | HIMA | RA | 0.1485 (0.0030) | 0.1908 (0.0049) | 0.2330 (0.0052) | 0.2793 (0.0071) |
|  |  | PSR | 0.1590 (0.0033) | 0.2029 (0.0053) | 0.2479 (0.0052) | 0.2971 (0.0073) |
|  |  | IPW | 0.1569 (0.0035) | 0.2006 (0.0054) | 0.2463 (0.0052) | 0.2937 (0.0077) |
|  |  | OW | 0.1559 (0.0030) | 0.2003 (0.0047) | 0.2453 (0.0047) | 0.2939 (0.0067) |
|  | mHIMA2 | RA | 0.1437 (0.0029) | 0.1832 (0.0043) | 0.2227 (0.0049) | 0.2673 (0.0067) |
|  |  | PSR | 0.1447 (0.0029) | 0.1885 (0.0045) | 0.2299 (0.0046) | 0.2799 (0.0066) |
|  |  | IPW | 0.1488 (0.0033) | 0.1915 (0.0051) | 0.2360 (0.0050) | 0.2835 (0.0076) |
|  |  | OW | 0.1480 (0.0029) | 0.1915 (0.0045) | 0.2353 (0.0046) | 0.2839 (0.0067) |
| n=500 | HIMA | RA | 0.1593 (0.0017) | 0.2033 (0.0028) | 0.2456 (0.0031) | 0.2979 (0.0035) |
|  |  | PSR | 0.1681 (0.0020) | 0.2121 (0.0033) | 0.2565 (0.0035) | 0.3082 (0.0040) |
|  |  | IPW | 0.1597 (0.0020) | 0.2041 (0.0032) | 0.2467 (0.0034) | 0.3000 (0.0041) |
|  |  | OW | 0.1596 (0.0017) | 0.2036 (0.0028) | 0.2459 (0.0030) | 0.2984 (0.0036) |
|  | mHIMA2 | RA | 0.1507 (0.0016) | 0.1917 (0.0027) | 0.2315 (0.0031) | 0.2793 (0.0037) |
|  |  | PSR | 0.1527 (0.0017) | 0.1962 (0.0027) | 0.2378 (0.0031) | 0.2891 (0.0035) |
|  |  | IPW | 0.1548 (0.0019) | 0.1989 (0.0031) | 0.2409 (0.0034) | 0.2926 (0.0040) |
|  |  | OW | 0.1547 (0.0017) | 0.1985 (0.0027) | 0.2401 (0.0031) | 0.2909 (0.0035) |

^a^ MT methods denote two different mediation test approaches, including HIMA Zhang et al. and modified HIMA2 Perera et al. (termed mHIMA2).

^b^ CONF methods denote different confounding adjustment methods.

^*^ RA denotes regression adjustment. PSR denotes propensity score regression adjustment. IPW denotes inverse probability weighting. OW denotes overlapping weighting.

Table S3. TPR and FDP for the four true mediators (M1–M4), $\rho=0.25,p=1000$.

| Sample size | MT method^a^ | CONF method^b^ | TPR | | | | Overall TPR | FDP |
| --- | --- | --- | --- | --- | --- | --- | --- | --- |
|  |  |  | M1 $\alpha\beta$= 0.16 | M2 $\alpha\beta$= 0.20 | M3 $\alpha\beta$= 0.25 | M4 $\alpha\beta$= 0.30 |  |  |
| n=300 | HIMA | RA | 0.5240 | 0.5360 | 0.7800 | 0.7660 | 0.6515 | 0.0053 |
|  |  | PSR | 0.4620 | 0.4840 | 0.7260 | 0.7000 | 0.5930 | 0.0025 |
|  |  | IPW | 0.3400 | 0.3800 | 0.5960 | 0.5920 | 0.4770 | 0.0031 |
|  |  | OW | 0.3760 | 0.3980 | 0.6260 | 0.6360 | 0.5090 | 0.0029 |
|  | mHIMA2 | RA | 0.6483 | 0.6547 | 0.8496 | 0.8538 | 0.7516 | 0.0160 |
|  |  | PSR | 0.8286 | 0.8306 | 0.9415 | 0.9355 | 0.8841 | 0.0754 |
|  |  | IPW | 0.7646 | 0.7626 | 0.8934 | 0.9034 | 0.8310 | 0.0630 |
|  |  | OW | 0.8233 | 0.8213 | 0.9277 | 0.9337 | 0.8765 | 0.0608 |
| n=500 | HIMA | RA | 0.8100 | 0.7960 | 0.9800 | 0.9780 | 0.8910 | 0.0006 |
|  |  | PSR | 0.7840 | 0.7700 | 0.9680 | 0.9660 | 0.8720 | 0.0011 |
|  |  | IPW | 0.6380 | 0.6340 | 0.8860 | 0.9040 | 0.7655 | 0.0007 |
|  |  | OW | 0.6900 | 0.6860 | 0.9260 | 0.9320 | 0.8085 | 0.0000 |
|  | mHIMA2 | RA | 0.8785 | 0.8623 | 0.9919 | 0.9899 | 0.9307 | 0.0070 |
|  |  | PSR | 0.9679 | 0.9519 | 1.0000 | 1.0000 | 0.9800 | 0.0761 |
|  |  | IPW | 0.9220 | 0.9220 | 0.9860 | 0.9840 | 0.9535 | 0.0624 |
|  |  | OW | 0.9660 | 0.9600 | 1.0000 | 0.9980 | 0.9810 | 0.0558 |

Table S4. Estimation results of mediation effects, expressed as Mean (MSE), $\rho=0.25,p=1000$.

| Sample size | MT method^a^ | CONF method^b^ | M1 $\alpha\beta$= 0.16 (MSE) | M2 $\alpha\beta$= 0.20 (MSE) | M3 $\alpha\beta$= 0.25 (MSE) | M4 $\alpha\beta$= 0.30 (MSE) |
| --- | --- | --- | --- | --- | --- | --- |
| n=300 | HIMA | RA | 0.1573 (0.0031) | 0.1997 (0.0046) | 0.2427 (0.0050) | 0.2869 (0.0063) |
|  |  | PSR | 0.1635 (0.0033) | 0.2056 (0.0050) | 0.2484 (0.0052) | 0.2953 (0.0065) |
|  |  | IPW | 0.1622 (0.0034) | 0.2064 (0.0056) | 0.2507 (0.0056) | 0.2993 (0.0076) |
|  |  | OW | 0.1620 (0.0031) | 0.2067 (0.0049) | 0.2502 (0.0051) | 0.2961 (0.0061) |
|  | mHIMA2 | RA | 0.1562 (0.0031) | 0.1971 (0.0043) | 0.2376 (0.0049) | 0.2794 (0.0063) |
|  |  | PSR | 0.1574 (0.0031) | 0.2032 (0.0048) | 0.2472 (0.0050) | 0.2892 (0.0063) |
|  |  | IPW | 0.1590 (0.0033) | 0.2043 (0.0054) | 0.2490 (0.0056) | 0.2938 (0.0075) |
|  |  | OW | 0.1586 (0.0031) | 0.2040 (0.0048) | 0.2479 (0.0051) | 0.2905 (0.0063) |
| n=500 | HIMA | RA | 0.1614 (0.0018) | 0.1991 (0.0028) | 0.2499 (0.0028) | 0.3030 (0.0037) |
|  |  | PSR | 0.1686 (0.0021) | 0.2051 (0.0031) | 0.2569 (0.0031) | 0.3119 (0.0043) |
|  |  | IPW | 0.1612 (0.0020) | 0.2000 (0.0032) | 0.2506 (0.0033) | 0.3043 (0.0042) |
|  |  | OW | 0.1614 (0.0018) | 0.1995 (0.0028) | 0.2505 (0.0028) | 0.3034 (0.0037) |
|  | mHIMA2 | RA | 0.1568 (0.0018) | 0.1941 (0.0027) | 0.2435 (0.0026) | 0.2934 (0.0036) |
|  |  | PSR | 0.1579 (0.0018) | 0.1991 (0.0029) | 0.2499 (0.0029) | 0.3005 (0.0038) |
|  |  | IPW | 0.1584 (0.0019) | 0.1989 (0.0033) | 0.2490 (0.0033) | 0.3007 (0.0042) |
|  |  | OW | 0.1587 (0.0018) | 0.1985 (0.0028) | 0.2489 (0.0028) | 0.2999 (0.0037) |

Table S5. TPR and FDP for the four true mediators (M1–M4), $\rho=0.5,p=1000$.

| Sample size | MT method^a^ | CONF method^b^ | TPR | | | | Overall TPR | FDP |
| --- | --- | --- | --- | --- | --- | --- | --- | --- |
|  |  |  | M1 $\alpha\beta$= 0.16 | M2 $\alpha\beta$= 0.20 | M3 $\alpha\beta$= 0.25 | M4 $\alpha\beta$= 0.30 |  |  |
| n=300 | HIMA | RA | 0.4840 | 0.4760 | 0.7740 | 0.7400 | 0.6185 | 0.0048 |
|  |  | PSR | 0.4000 | 0.4220 | 0.7040 | 0.6780 | 0.5510 | 0.0018 |
|  |  | IPW | 0.3300 | 0.3180 | 0.5540 | 0.5540 | 0.4390 | 0.0034 |
|  |  | OW | 0.3460 | 0.3540 | 0.6140 | 0.5960 | 0.4775 | 0.0010 |
|  | mHIMA2 | RA | 0.6626 | 0.6646 | 0.8753 | 0.8507 | 0.7633 | 0.0490 |
|  |  | PSR | 0.8101 | 0.8141 | 0.9313 | 0.9313 | 0.8717 | 0.0944 |
|  |  | IPW | 0.7399 | 0.7157 | 0.8871 | 0.8629 | 0.8014 | 0.0767 |
|  |  | OW | 0.7956 | 0.8056 | 0.9339 | 0.9058 | 0.8602 | 0.0798 |
| n=500 | HIMA | RA | 0.8040 | 0.8440 | 0.9720 | 0.9580 | 0.8945 | 0.0017 |
|  |  | PSR | 0.7720 | 0.7980 | 0.9640 | 0.9620 | 0.8740 | 0.0017 |
|  |  | IPW | 0.6340 | 0.6500 | 0.8860 | 0.9000 | 0.7675 | 0.0007 |
|  |  | OW | 0.6960 | 0.7080 | 0.9240 | 0.9320 | 0.8150 | 0.0006 |
|  | mHIMA2 | RA | 0.9058 | 0.9218 | 0.9880 | 0.9840 | 0.9499 | 0.0400 |
|  |  | PSR | 0.9660 | 0.9720 | 0.9940 | 0.9960 | 0.9820 | 0.0878 |
|  |  | IPW | 0.9280 | 0.9260 | 0.9780 | 0.9860 | 0.9545 | 0.0760 |
|  |  | OW | 0.9520 | 0.9580 | 0.9960 | 0.9920 | 0.9745 | 0.0706 |

Table S6. Estimation results of mediation effects, expressed as Mean (MSE), $\rho=0.5,p=1000$.

| Sample size | MT method^a^ | CONF method^b^ | M1 $\alpha\beta$= 0.16 (MSE) | M2 $\alpha\beta$= 0.20 (MSE) | M3 $\alpha\beta$= 0.25 (MSE) | M4 $\alpha\beta$= 0.30 (MSE) |
| --- | --- | --- | --- | --- | --- | --- |
| n=300 | HIMA | RA | 0.1581 (0.0031) | 0.1964 (0.0049) | 0.2438 (0.0051) | 0.2876 (0.0067) |
|  |  | PSR | 0.1598 (0.0033) | 0.1988 (0.0049) | 0.2463 (0.0051) | 0.2921 (0.0069) |
|  |  | IPW | 0.1613 (0.0037) | 0.2009 (0.0055) | 0.2477 (0.0060) | 0.2935 (0.0072) |
|  |  | OW | 0.1599 (0.0032) | 0.1999 (0.0049) | 0.2485 (0.0051) | 0.2933 (0.0065) |
|  | mHIMA2 | RA | 0.1590 (0.0032) | 0.1975 (0.0050) | 0.2484 (0.0051) | 0.2891 (0.0070) |
|  |  | PSR | 0.1599 (0.0033) | 0.2025 (0.0052) | 0.2554 (0.0055) | 0.2952 (0.0072) |
|  |  | IPW | 0.1606 (0.0037) | 0.2026 (0.0058) | 0.2522 (0.0060) | 0.2932 (0.0077) |
|  |  | OW | 0.1589 (0.0033) | 0.2011 (0.0051) | 0.2524 (0.0052) | 0.2929 (0.0070) |
| n=500 | HIMA | RA | 0.1577 (0.0018) | 0.2003 (0.0026) | 0.2493 (0.0030) | 0.2975 (0.0035) |
|  |  | PSR | 0.1630 (0.0019) | 0.2022 (0.0027) | 0.2521 (0.0032) | 0.3022 (0.0036) |
|  |  | IPW | 0.1581 (0.0020) | 0.2008 (0.0028) | 0.2517 (0.0034) | 0.2994 (0.0037) |
|  |  | OW | 0.1584 (0.0018) | 0.2009 (0.0026) | 0.2501 (0.0030) | 0.2980 (0.0035) |
|  | mHIMA2 | RA | 0.1587 (0.0018) | 0.1999 (0.0026) | 0.2506 (0.0030) | 0.2952 (0.0037) |
|  |  | PSR | 0.1590 (0.0018) | 0.2040 (0.0028) | 0.2562 (0.0032) | 0.3013 (0.0039) |
|  |  | IPW | 0.1580 (0.0020) | 0.2015 (0.0029) | 0.2547 (0.0036) | 0.2998 (0.0040) |
|  |  | OW | 0.1583 (0.0018) | 0.2016 (0.0027) | 0.2530 (0.0031) | 0.2983 (0.0038) |

Table S7. TPR and FDP for the four true mediators (M1–M4), $\rho=0.75,p=1000$.

| Sample size | MT method^a^ | CONF method^b^ | TPR | | | | Overall TPR | FDP |
| --- | --- | --- | --- | --- | --- | --- | --- | --- |
|  |  |  | M1 $\alpha\beta$= 0.16 | M2 $\alpha\beta$= 0.20 | M3 $\alpha\beta$= 0.25 | M4 $\alpha\beta$= 0.30 |  |  |
| n=300 | HIMA | RA | 0.4500 | 0.3580 | 0.6400 | 0.7420 | 0.5475 | 0.0126 |
|  |  | PSR | 0.4020 | 0.3560 | 0.6380 | 0.6900 | 0.5215 | 0.0123 |
|  |  | IPW | 0.3240 | 0.2700 | 0.5180 | 0.5800 | 0.4230 | 0.0117 |
|  |  | OW | 0.3400 | 0.2940 | 0.5520 | 0.6240 | 0.4525 | 0.0131 |
|  | mHIMA2 | RA | 0.7919 | 0.7980 | 0.9293 | 0.9434 | 0.8657 | 0.0946 |
|  |  | PSR | 0.7776 | 0.7936 | 0.9419 | 0.9499 | 0.8657 | 0.0977 |
|  |  | IPW | 0.7264 | 0.7445 | 0.8934 | 0.8833 | 0.8119 | 0.0819 |
|  |  | OW | 0.7811 | 0.7771 | 0.9378 | 0.9378 | 0.8584 | 0.0782 |
| n=500 | HIMA | RA | 0.8180 | 0.7360 | 0.9260 | 0.9600 | 0.8600 | 0.0023 |
|  |  | PSR | 0.7780 | 0.7100 | 0.9060 | 0.9440 | 0.8345 | 0.0030 |
|  |  | IPW | 0.6340 | 0.6300 | 0.8220 | 0.8660 | 0.7380 | 0.0020 |
|  |  | OW | 0.6880 | 0.6720 | 0.8740 | 0.9200 | 0.7885 | 0.0025 |
|  | mHIMA2 | RA | 0.9559 | 0.9599 | 0.9960 | 0.9940 | 0.9765 | 0.0863 |
|  |  | PSR | 0.9620 | 0.9600 | 1.0000 | 0.9940 | 0.9790 | 0.0948 |
|  |  | IPW | 0.9360 | 0.9340 | 0.9800 | 0.9800 | 0.9575 | 0.0713 |
|  |  | OW | 0.9560 | 0.9580 | 0.9980 | 0.9960 | 0.9770 | 0.0682 |

Table S8. Estimation results of mediation effects, expressed as Mean (MSE), $\rho=0.75,p=1000$.

| Sample size | MT method^a^ | CONF method^b^ | M1 $\alpha\beta$= 0.16 (MSE) | M2 $\alpha\beta$= 0.20 (MSE) | M3 $\alpha\beta$= 0.25 (MSE) | M4 $\alpha\beta$= 0.30 (MSE) |
| --- | --- | --- | --- | --- | --- | --- |
| n=300 | HIMA | RA | 0.1852 (0.0079) | 0.2322 (0.0105) | 0.2880 (0.0120) | 0.3130 (0.0106) |
|  |  | PSR | 0.1800 (0.0070) | 0.2202 (0.0077) | 0.2793 (0.0102) | 0.3055 (0.0097) |
|  |  | IPW | 0.1856 (0.0081) | 0.2331 (0.0102) | 0.2901 (0.0124) | 0.3167 (0.0116) |
|  |  | OW | 0.1842 (0.0074) | 0.2327 (0.0098) | 0.2885 (0.0116) | 0.3154 (0.0108) |
|  | mHIMA2 | RA | 0.1729 (0.0047) | 0.2009 (0.0063) | 0.2668 (0.0073) | 0.3090 (0.0093) |
|  |  | PSR | 0.1718 (0.0048) | 0.2074 (0.0067) | 0.2745 (0.0080) | 0.3154 (0.0096) |
|  |  | IPW | 0.1694 (0.0049) | 0.2022 (0.0067) | 0.2675 (0.0081) | 0.3091 (0.0102) |
|  |  | OW | 0.1682 (0.0046) | 0.2012 (0.0063) | 0.2662 (0.0074) | 0.3082 (0.0091) |
| n=500 | HIMA | RA | 0.1729 (0.0034) | 0.2091 (0.0039) | 0.2629 (0.0059) | 0.3020 (0.0051) |
|  |  | PSR | 0.1750 (0.0037) | 0.2068 (0.0041) | 0.2625 (0.0065) | 0.3025 (0.0054) |
|  |  | IPW | 0.1712 (0.0033) | 0.2085 (0.0040) | 0.2607 (0.0057) | 0.3047 (0.0057) |
|  |  | OW | 0.1705 (0.0032) | 0.2075 (0.0038) | 0.2597 (0.0053) | 0.3034 (0.0052) |
|  | mHIMA2 | RA | 0.1724 (0.0026) | 0.2032 (0.0037) | 0.2611 (0.0048) | 0.3040 (0.0058) |
|  |  | PSR | 0.1733 (0.0027) | 0.2079 (0.0039) | 0.2690 (0.0054) | 0.3105 (0.0061) |
|  |  | IPW | 0.1709 (0.0028) | 0.2033 (0.0039) | 0.2610 (0.0052) | 0.3048 (0.0064) |
|  |  | OW | 0.1701 (0.0026) | 0.2027 (0.0036) | 0.2604 (0.0049) | 0.3035 (0.0059) |

Table S9. Summary of the selected CpGs mediators with a %TE>5 by the HIMA models.

| Method^*^ | CpG | Chrom | Gene | $\hat{\alpha}$ | $\hat{\beta}$ | %TE | $p$-value |
| --- | --- | --- | --- | --- | --- | --- | --- |
| HIMA-RA | cg06202778 | 4 | HOPX | 0.0172 | -0.0777 | 5.7997 | 0.0001 |
|  | cg00305629 | 9 | DFNB31 | 0.0126 | -0.0955 | 5.2236 | 0.0011 |
|  | cg14057303 | 9 | MIR548Q | 0.0153 | -0.0907 | 6.0309 | 0.0009 |
|  | cg06040872 | 17 | CCL18 | 0.0157 | -0.1261 | 8.5816 | 0.0014 |
|  | cg16893868 | 19 | LILRA2 | -0.0155 | 0.1001 | 6.7437 | 0.0001 |
| HIMA-PSR | cg13917614 | 17 | CNP | 0.0230 | -0.2247 | 22.4717 | 0.0038 |
|  | cg03048488 | 1 | RP3-467K16.4 | 0.0135 | -0.1140 | 6.7044 | 0.0082 |
|  | cg03164561 | 2 | NMUR1 | 0.0149 | -0.1652 | 10.6921 | 0.0030 |
|  | cg20657709 | 2 | BRE | 0.0160 | -0.1088 | 7.5794 | 0.0212 |
|  | cg03605454 | 4 | RP11-526A4.1 | 0.0154 | -0.1049 | 7.0143 | 0.0363 |
|  | cg13789303 | 6 | FOXO3 | -0.0219 | 0.0631 | 6.0059 | 0.0328 |
|  | cg09529165 | 17 | - | 0.0198 | -0.2335 | 20.1310 | <0.0001 |
|  | cg01500140 | 19 | LIM2 | 0.0157 | -0.2218 | 15.1334 | 0.0221 |
|  | cg16893868 | 19 | LILRA2 | -0.0155 | 0.1293 | 8.7093 | 0.0038 |
| HIMA-IPW | cg13917614 | 17 | CNP | 0.0230 | -0.0796 | 7.9180 | 0.0044 |
|  | cg06202778 | 4 | HOPX | 0.0173 | -0.0747 | 5.5865 | 0.0129 |
|  | cg11100450 | 6 | EXOC2 | 0.0169 | -0.0771 | 5.6286 | 0.0058 |
|  | cg01500140 | 19 | LIM2 | 0.0158 | -0.0908 | 6.2079 | 0.0194 |
|  | cg16893868 | 19 | LILRA2 | -0.0157 | 0.0945 | 6.4136 | 0.0044 |
| HIMA-OW | cg13917614 | 17 | CNP | 0.0230 | -0.0796 | 7.9641 | 0.0050 |
|  | cg06202778 | 4 | HOPX | 0.0172 | -0.0747 | 5.5706 | 0.0135 |
|  | cg11100450 | 6 | EXOC2 | 0.0167 | -0.0771 | 5.5793 | 0.0058 |
|  | cg01500140 | 19 | LIM2 | 0.0157 | -0.0908 | 6.1951 | 0.0202 |
|  | cg16893868 | 19 | LILRA2 | -0.0155 | 0.0945 | 6.3669 | 0.0050 |

^*^ Method denotes the combination of the HIMA approach and different confounding adjustment methods. Such as HIMA-OW denotes the HIMA method with overlapping weighting.

- No related genes were found.
